# Role of L-Type Amino Acid Transporter 1 (LAT1) for the Selective Cytotoxicity of Sesamol in Human Melanoma Cells

**DOI:** 10.3390/molecules24213869

**Published:** 2019-10-27

**Authors:** Tarapong Srisongkram, Natthida Weerapreeyakul, Jussi Kärkkäinen, Jarkko Rautio

**Affiliations:** 1 Graduate School (in the program of Research and Development in Pharmaceuticals), Faculty of Pharmaceutical Sciences, Khon Kaen University, Khon Kaen 40002, Thailand; tarapong.sri@gmail.com; 2 Division of Pharmaceutical Chemistry, Faculty of Pharmaceutical Sciences, Khon Kaen University, Khon Kaen 40002, Thailand; 3 Human High Performance and Health Promotion (HHP&HP) Research Institute, Khon Kaen University, Khon Kaen 40002, Thailand; 4 School of Pharmacy, University of Eastern Finland, Kuopio 70211, Finland; jussi.karkkainen@uef.fi (J.K.); jarkko.rautio@uef.fi (J.R.)

**Keywords:** *Sesamum indicum*, Pedaliaceae, sesamol, L-type amino acid transporter 1, melanoma, uptake transport, selective cytotoxicity

## Abstract

Sesamol is effective against melanoma cells with less damage to normal cells. The underlying selective cytotoxicity of sesamol in melanoma vs. non-cancerous cells is undefined. Melanoma cells differ from normal cells by over-expression of the L-type amino acid transporter 1 (LAT1). We sought to clarify the transport mechanism on selective cytotoxicity of sesamol in melanoma cells. A human melanoma cell line (SK-MEL-2) and African monkey epithelial cell line (Vero) were used to study the cellular uptake and cytotoxicity of sesamol. The intracellular concentration of sesamol was quantified by UV-HPLC. The cytotoxicity was determined by neutral red uptake assay. Sesamol showed a higher distribution volume and uptake clearance in SK-MEL-2 than Vero cells. Sesamol was distributed by both carrier-mediated and passive transport by having greater carrier-mediated transport into SK-MEL-2 cells than Vero cells. Higher mRNA expression and function of LAT1 over LAT2 were evident in SK-MEL-2 cells compared to Vero cells. Sesamol uptake and sesamol cytotoxicity were inhibited by the LAT1 inhibitor, suggesting LAT1 had a role in sesamol transport and its bioactivity in melanoma. The LAT1-mediated transport of sesamol is indicative of how it engages cytotoxicity in melanoma cells with promising therapeutic benefits.

## 1. Introduction

Most small molecules are transported across cell membranes via passive transport and/or carrier-mediated transport [1,2,3,4]. Carrier-mediated transporters are key determinants for pharmacokinetic, safety, and efficacy profiles of small molecules [4]. Carrier-mediated proteins overexpressed in melanoma cells include L-type amino acid transporter 1 (LAT1/SLC7A5) [5,6,7], L-type amino acid transporter 2 (LAT2/SLC7A8) [5,6,7], alanine-serine-cysteine transporter 2 (ASCT2/SLC1A5) [5,6,7], and glucose uptake transporter 1 (GLUT1) [8]. LAT1 is more highly expressed in skin cancer than other types of cancer [5]. LAT1 is thus used as a biomarker for disease progression in malignant melanoma patients [6].

Sesame seed oil from *Sesamum indicum* L. (Pedaliaceae) has long been used to preserve skin moisture because it is rich in anti-oxidants (i.e., sesamin, sesamolin and sesamol) [9,10]. Sesamol also possesses skin lightening, skin protective [11,12,13,14,15]], anti-melasma [15], and selective anti-proliferative effects, as well as inducing apoptotic cell death against melanoma SK-MEL-2 cells without damaging non-cancerous Vero cells [13,14,16]. The selective cytotoxicity of sesamol has been reported for adenocarcinoma lung cancer, colon cancer, and cervical cancer cell lines [16,17,18], and in malignant melanoma and hepatocellular carcinoma in mice [19,20]. Sesamol has been shown to have potential for treatment of melanoma; however, the lower lipophilicity (logP 1.29) of sesamol seems to hinder its uptake and consequently its action in the cells.

As LAT1 is predominantly expressed in melanoma cells, our aim was to investigate LAT1 vis-à-vis its role in intracellular uptake of sesamol and the antiproliferative effect against melanoma cells. SK-MEL-2 and Vero cells were used for melanoma and non-cancerous epithelial renal cell models, respectively. The information obtained herein regarding sesamol distribution in melanoma and epithelial cell models will guide its clinical use for predicting the safety and effectiveness against high or low LAT1 expression prior to pre-clinical animal experimentation.

## 2. Results

### 2.1. Sesamol Distribution into SK-MEL-2 and Vero Cells

**Figure 1:**
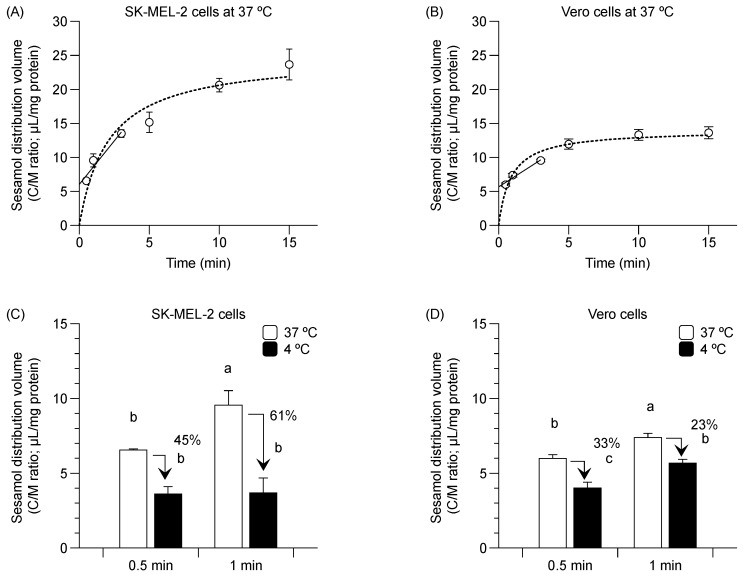
Time-course relationship of sesamol distribution in SK-MEL-2 and Vero cells. (**A**) Distribution volume of sesamol uptake at 37 °C in SK-MEL-2 cells and (**B**) in Vero cells. Linear time-course uptake fitted by linear regression least square (─) and equilibrium uptake by non-linear regression least square models (···). Sesamol distribution was measured at 4 °C (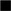) and 37 °C (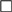) in (**C**) SK-MEL-2 and (**D**) Vero cells. Data points represent triplicates and are expressed as mean ± standard deviation. Different letters between bars indicate significant differences between samples (*p* < 0.05).

Sesamol distribution into the SK-MEL-2 and Vero cells at 37 °C occurred in a time-dependent manner (i.e., from 30 s to 3 min) (Figure 1A,B, solid line). The respective estimation of the initial distribution volume and initial uptake clearance in SK-MEL-2 cells was 6.0 ± 0.4 µL/mg protein and 2.6 ± 0.1 µL/min/mg protein. The respective calculated values of the initial distribution volume and initial uptake clearance in Vero cells was 5.7 ± 0.4 µL/mg protein and 1.3 ± 0.2 µL/min/mg protein. Sesamol uptake reached a steady state at approximately 10 min in the SK-MEL-2 cells (Figure 1A, dotted line) and in about 5 min in the Vero cells (Figure 1B, dotted line).

Sesamol distribution was also conducted at 4 °C. Sesamol uptake in SK-MEL-2 cells was suppressed by 45% and 61% at 4 °C (compared to 37 °C) at 30 s and 1 min, respectively (Figure 1C). A similar reduction in the distribution volume was observed in the Vero cells albeit to a lesser extent (by 33% and 23% at 30 s and 1 min, respectively) decrease in sesamol uptake compared with that of 37 °C (Figure 1D).

The greater initial distribution volume and uptake clearance of sesamol in the SK-MEL-2 cells over against Vero cells suggests that there is higher sesamol uptake in the SK-MEL-2 cells than in the Vero cells. The decreased sesamol distribution at 4 °C in both cells confirms that sesamol was taken up via carrier-mediated transportation and passive diffusion.

### 2.2. Uptake Kinetics of Sesamol in SK-MEL-2 and Vero Cells

**Figure 2:**
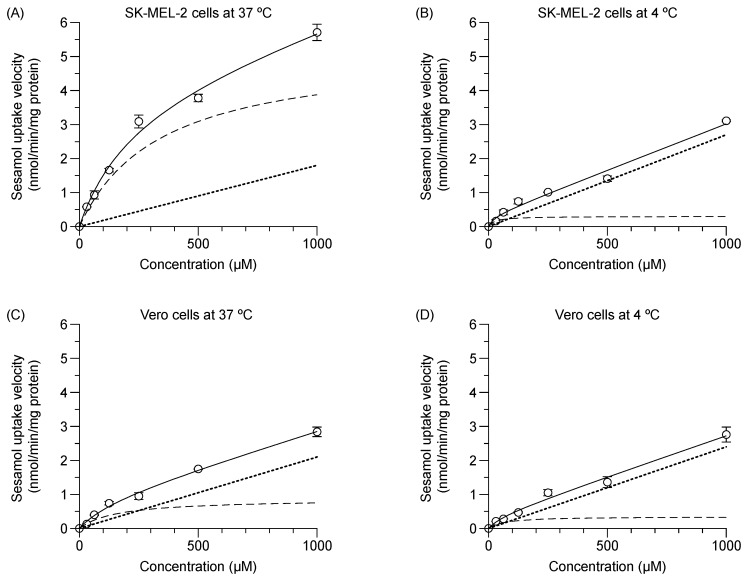
Concentration dependence of sesamol influx mechanism at 37 °C and 4 °C in SK-MEL-2 and Vero cells over one minute. (**A**,**B**) Sesamol uptake at 37 °C and 40 °C in SK-MEL-2 and (**C**,**D**) sesamol uptake at 37 °C and 4 °C in Vero cells, respectively. Experimental total transport (─), predicted carrier mediated transport (---), and passive transport (···) are depicted. Data are expressed as mean ± standard deviation of triplicates with two analytical runs.

The experimental total initial transport of sesamol (1 min) into the SK-MEL-2 and Vero cells at 37 °C and 4 °C were concentration-dependent (Figure 2); facilitating calculation of the predicted values of (1) carrier-mediated transport or saturable kinetics, and (2) passive transport or non-saturable kinetics. In SK-MEL-2 cells (Figure 2A), the saturable kinetic of sesamol represented carrier-mediated transport with *V*_max_ and *K*_m_ values of 4.2 ± 0.1 nmol/min/mg protein and 224.6 ± 19.3 µM, respectively. The non-saturable kinetics constituted passive diffusion of sesamol with a *P*_d_ value of 2.3 ± 0.2 µL/min/mg protein (Table 1). At 37 °C, the uptake clearance of the carrier-mediated transport (*V*_max_/*K*_m_) was 18.5 µL/min/mg protein, while total uptake clearance was 18.5 + 2.3 = 20.8 µL/min/mg protein. The carrier-mediated transport (18.5 µL/min/mg protein) was 89% of total uptake clearance of the uptake process (20.8 µL/min/mg protein).

At 4 °C (Figure 2B), carrier-mediated transport was clearly suppressed. The calculated *V*_max_ and *K*_m_ values were significantly decreased from those at 37 °C (*p* < 0.05); while the *P*_d_ value was significantly increased (*p* < 0.05) compared to those at 37 °C. The results suggest that intracellular uptake of sesamol at 4 °C was predominantly by passive diffusion.

In the Vero cells at 37 °C (Figure 2C), the respective calculated value for *V*_max_ and *K*_m_ was 0.9 ± 0.0 nmol/min/mg protein and 159.9 ± 18.4 µM, while the *P*_d_ value was 2.1 ± 0.1 µL/min/mg protein. The uptake clearance of carrier-mediated transport of sesamol (*V*_max_/*K*_m_) was 5.4 µL/min/mg protein or 72% of the total uptake clearance of the uptake process in the Vero cells (7.5 µL/min/mg protein). In the Vero cells at 4 °C (Figure 2D), the respective *V*_max_ and *K*_m_ values were significantly reduced (*p* < 0.05), while the *P*_d_ value was nominally increased compared with the values at 37 °C. Remarkably, the carrier-mediated uptake clearance of sesamol at 37 °C in the SK-MEL-2 cells was 3.4-fold greater than that of the Vero cells. Low passive diffusion of sesamol at 37 °C was evident in the SK-MEL-2 cells indicating its minor role.

### 2.3. LAT1 Activity and Equilibrium Distribution of Sesamol

**Figure 3:**
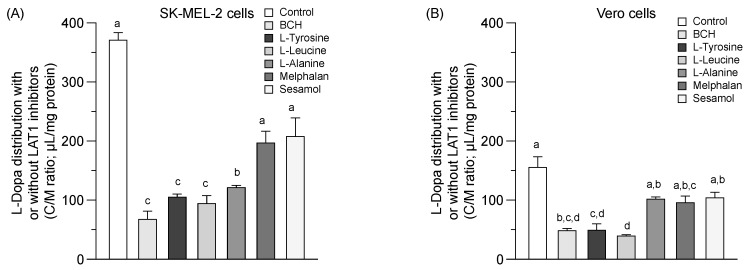

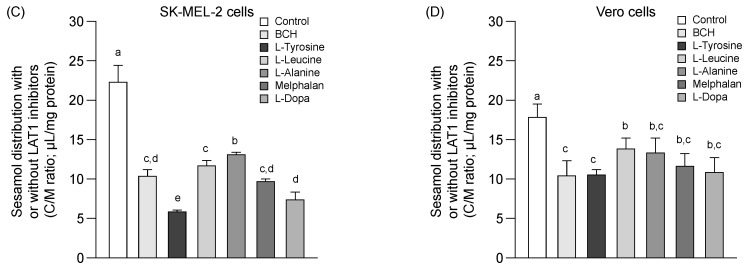
Competitive distribution volume of L-dopa and sesamol by LAT1 substrates/inhibitor in SK-MEL-2 and Vero cells. The competitive distribution of L-dopa in (**A**) SK-EML-2 and (**B**) Vero cells. The competitive distribution of sesamol into (**C**) SK-MEL-2 and (**D**) Vero cells, respectively. Data are expressed as mean ± standard deviation of triplicates with two analytical runs. Different letters in the same column indicate significant differences between samples (*p* < 0.05).

A greater L-dopa (or control) uptake into the SK-MEL-2 cells than the Vero cells at 37 °C was observed (Figure 3A,B). L-dopa uptake was significantly inhibited under the various conditions studied in both cells (*p* < 0.05). Sesamol and melphalan was non-significantly decreased the uptake of L-dopa in both the SK-MEL-2 and Vero cells compared to the control. LAT1 function seemed higher in the SK-MEL-2 cells than in the Vero cells. The inhibition of L-dopa distribution by sesamol in both cells suggests that sesamol may act as either a LAT1 inhibitor or a LAT1 substrate.

The distribution of sesamol (Figure 3C,D, control) was evaluated at the time of uptake equilibrium (10 min) (Figure 1A) to determine the feasibility of sesamol distribution via LAT1. Sesamol was distributed into the SK-MEL-2 cells more than into the Vero cells (Figure 3C,D & Table 2). The distribution of sesamol was significantly inhibited under all conditions studied (*p* < 0.05) in the SK-MEL-2 cells. This inhibition effect was more extensive in the SK-MEL-2 cells than in the Vero cells. The respective competitive uptake between sesamol and known LAT1 substrates (L-leucine, L-tyrosine, melphalan, and L-dopa) and LAT1 inhibitor (BCH) was higher in the SK-MEL-2 cells than in the Vero cells. These results indicate that sesamol might act as the LAT1 substrate; however, sesamol uptake also inhibited by L-alanine (with its low affinity to LAT1 and high affinity to LAT2), indicating the possibility of sesamol being taken up by carrier-proteins other than LAT1 (e.g., LAT2). More research is needed to define the specific transporter protein attributable to intracellular sesamol uptake was further performed.

### 2.4. Inhibition of Sesamol Uptake Kinetics in SK-MEL-2 and Vero Cells

**Figure 4:**
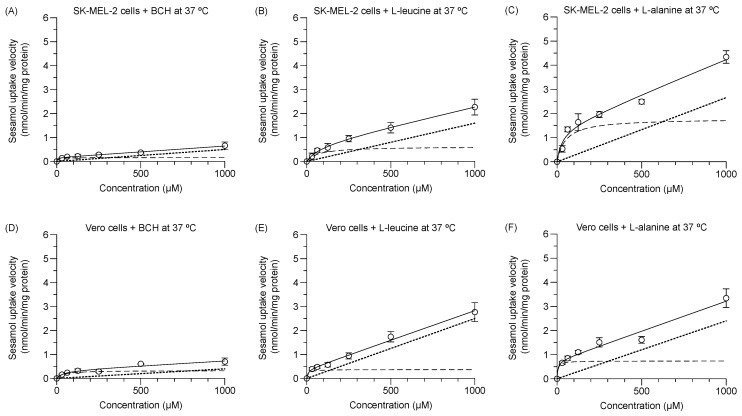
Concentration dependence of sesamol influx mechanism at 37 °C in presence of LAT1 inhibitor and LAT1 substrates in SK-MEL-2 and Vero cells. Kinetics of sesamol influx to SK-MEL-2 cells in presence of (**A**) 1 mM BCH, (**B**) 2 mM L-leucine, and (**C**) 2 mM L-alanine. Kinetics of sesamol influx to Vero cells in presence of (**D**) 1 mM BCH, (**E**) 2 mM L-leucine, and (**F**) 2 mM L-alanine. Experimental total transport (─), predicted carrier-mediated transport (---), and passive transport (···) are depicted. Data are expressed as mean ± standard deviation of triplicates with two analytical runs.

The respective experimental total transport of sesamol in the SK-MEL-2 and Vero cells at 37 °C with either the LAT1 inhibitor (1 mM BCH) or high and low affinity LAT1 substrates (i.e., 2 mM L-leucine and 2 mM L-alanine) was concentration-dependent (Figure 4A–F). The predicted carrier-mediated transport (*V*_max_ and *K*_m_; dashed line) and predicted passive transport (*P*_d_, dotted line) were calculated. The *V*_max_ and *P*_d_ values of sesamol were decreased under different conditions (Table 3) in both cells. The decreased degree of these values was higher in the SK-MEL-2 cells than in the Vero cells. The *K*_m_ values of sesamol in the SK-MEL-2 cells was decreased by BCH more than by either L-alanine or L-leucine. Meanwhile, the *K*_m_ values of sesamol in Vero cells were decreased by L-alanine more than by L-leucine and BCH. The reduction of both *V*_max_ and *K*_m_ in the presence of competitive substrates/inhibitor were largely affected by the rate of transporter re-configuration from endo- to exo-facially orientation [21]. In our experiment, the LAT1 substrates/inhibitor was preloaded into the cells prior to adding the sesamol solution, so it slowed the rate of LAT1 re-configuration from the preloaded substrate/inhibitor; explaining why both *V*_max_ and *K*_m_ of sesamol were reduced. The reduction of *V*_max_, *P*_d_, and *K*_m_ of sesamol under each condition compared to sesamol alone indicated that the amino acid transporter is responsible for the carrier-mediated transport of sesamol in these cells. Moreover, the reduction of *V*_max_ of sesamol under L-leucine and BCH was significantly greater than L-alanine, indicating that LAT1 has a more critical role in sesamol uptake more than LAT2 in SK-MEL-2 cells.

### 2.5. Anti-Proliferation in SK-MEL-2 and Vero Cells

**Figure 5:**
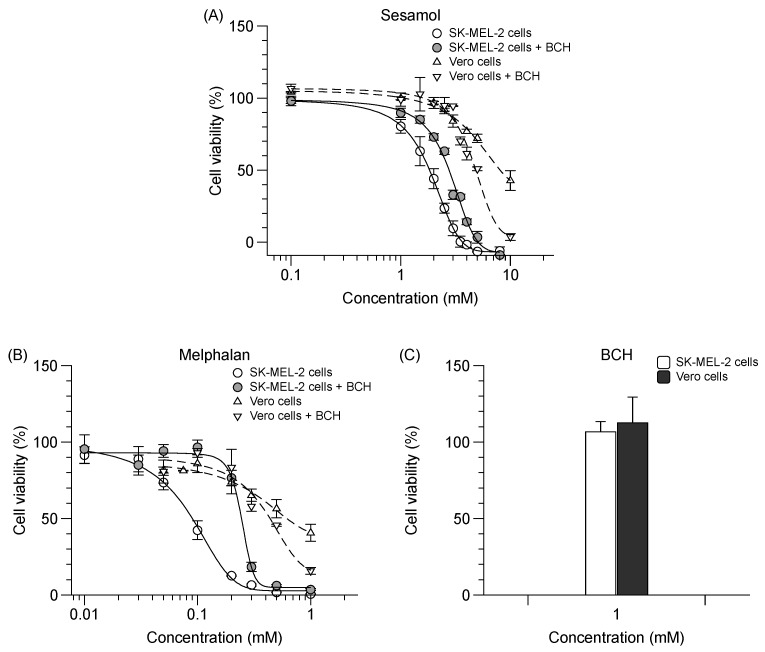
Effect of sesamol, melphalan and BCH on % cell viability in SK-MEL-2 and Vero cells. Antiproliferative effect of (**A**) sesamol and (**B**) melphalan in SK-MEL-2 cells (─) with (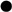) or without 1 mM BCH (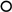); and in Vero cells (---) with (▽) or without 1 mM BCH (△). (**C**) Percent cytotoxicity exhibited by BCH in both cells. Sigmoidal curved fitted to calculate the IC_50_. Data are expressed as mean ± standard deviation of three to six replicates.

Sesamol possesses an antiproliferative effect on SK-MEL-2 cells (IC_50_ value of 2.0 ± 0.1 mM), albeit less potent than that of melphalan, which is a chemotherapeutic drug and a specific LAT1 substrate (IC_50_ value of 0.1 ± 0.0 mM). In the presence of BCH, the antiproliferative effect of sesamol and melphalan on SK-MEL-2 cells was decreased (Figure 5A,B) (*p* < 0.05). BCH (1 mM) exerted no cytotoxicity on both cell types as compared to untreated cells (Figure 5C). There was, however, a discrepancy due to the increased antiproliferative effect of sesamol and melphalan in the presence of BCH in Vero cells. Sesamol was selective for SK-MEL-2 cells, but to a lesser degree than melphalan (Table 4). The respective antiproliferative effect and selectivity index of sesamol vis-à-vis SK-MEL-2 cells was decreased when LAT1 function was inhibited by BCH (Table 4).

### 2.6. mRNA Expression of LAT1 Protein in SK-MEL-2 and Vero Cells

**Figure 6:**
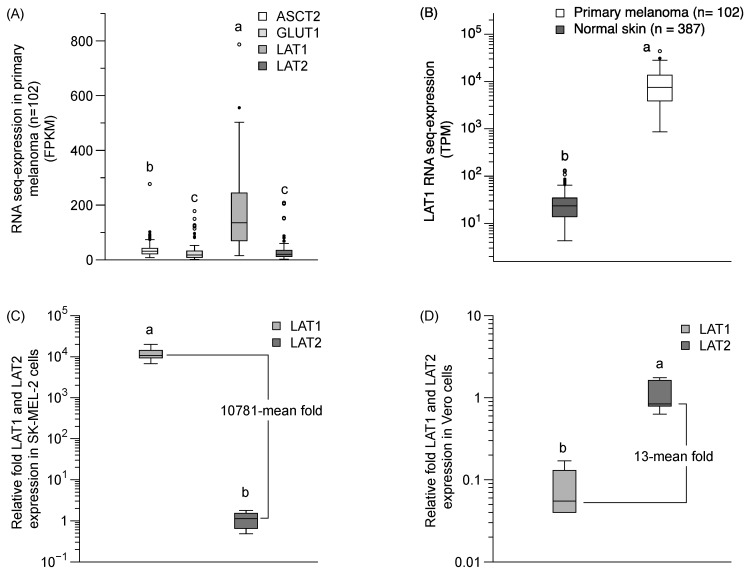
LAT1 expression in primary melanoma and in SK-MEL-2 and Vero cells. (**A**) LAT1 expression in primary melanoma from the Protein Atlas project bioinformatics database (https://www.proteinatlas.org). (**B**) Comparison of LAT1 expression between primary melanoma and normal skin. Data obtained from Protein Atlas project (https://www.proteinatlas.org). FPKM and TPM is stand for fragments per kilobase of exon per million reads and transcripts per million, respectively. The relative fold of mRNA qPCR analysis of LAT1 and LAT2 expression (**C**) in SK-MEL-2 cells and (**D**) in Vero cells. beta-Actin was used as a housekeeping gene. Each group represented the median (min–max) of six replications. Different letters (a, b) indicate significant differences between samples (*p* < 0.05).

We performed a qPCR analysis of LAT1 and LAT2 mRNA expression and bioinformatics analysis of a LAT1 expression in primary melanoma and normal skin, using from the Protein Atlas project (https://www.proteinatlas.org) [22]. The LAT1 in a primary melanoma is predominantly expressed over LAT2, ASCT2, and GLUT1 (Figure 6A). The LAT1 expression in primary melanoma was also significantly higher than in normal skin tissue (Figure 6B). The relative mRNA expression by qPCR revealed the predominant expression of the LAT1 protein (10781-mean fold) compared to the LAT2 protein in SK-MEL-2 cells (Figure 6C), and a lower level of LAT1 protein in normal cells (13-mean fold) than LAT2 protein in the Vero cells (Figure 6D). Our results confirmed a higher expression of LAT1 than LAT2 proteins in SK-MEL-2 cells, and a higher expression of LAT2 than LAT1 proteins in Vero cells—clearly supporting the notion that LAT1 has a significant role in sesamol uptake in the SK-MEL-2 cells.

## 3. Discussion

Melanoma is a complex disease involving multi mutation genes and displays the highest mutation among other cancer types [23]. Melanoma leads mortality cases by more than 10% of new cases in each year [24]. The multi mutation genes of melanoma patients reduce survival rates [25]. Despite new targeted therapy and immunotherapy, the combination of these two approaches has given rise to more adverse events [26]. Recently, LAT1 was introduced as a therapeutic target for treatment of melanoma [6,7,27], but the role of LAT1 in the transport of effective molecules into the melanoma cells has not been explored sufficiently.

LAT1 belongs to the L-type amino acid transporter (LAT) family [28] with broad structural requirements vis-à-vis LAT1 substrates. The shared common functional groups for LAT1 substrates are amino groups and hydrophilic meta-substituted aromatic rings (or hydrophobic side chains) [28,29,30,31,32]. Both the amino and carboxylic acid functional groups are mooted to be essential for transporter recognition [33], but some carboxylic esters [29] or carbonyl oxygens and alkoxy oxygens [31] could be taken up via LAT1 [29], indicating that the carboxyl functional group may not be essential [30,31,32]. The favorable stereoselectivity is L-amino acids, however, D-amino acids may also be taken up [29].

Three pocket sites for LAT1 substrates have been proposed [29]. Pocket A was bound with a hydrophilic hydroxyl group substitution at the meta-position of the aromatic ring. Pocket B was bound with a hydrophobic side chain; not necessarily a planar or π-stacking interaction. Pocket C was fitted to an alpha-carbon group, an amino group, and a carboxylic group. Pocket A and B needed to be occupied by the LAT1 substrate while pocket C could remain available [29]. The hydrophilic aromatic ring of sesamol seems to be key, allowing for a more favorable fit with pocket A and B; explaining why sesamol is susceptible to LAT1 or its being the LAT1 substrate.

Additional information to support the idea that sesamol is a LAT1 substrate was conducted by docking sesamol with LAT1 using a crystal structure of LAT1 protein from the databank (http://www.rcsb.org/) (PDB ID: 6IRT). The binding affinity was calculated by PyRx via autodock ViNA module [34]. The lowest binding affinity of sesamol with LAT1 was −4.7 kcal/mol (data not shown). The hydroxyl group of sesamol formed hydrogen bonds with serine 307, glutamic acid 309, and lysine 158 of LAT1 transmembrane and the oxygen in the benzodioxol ring formed hydrogen bonds with arginine 535 of 4F2hc domain (Figure S1).

LAT1 activity or expression can vary in different human cells or organs (i.e., being (a) high in the skeletal muscle and brain, (b) absent in the testis and stomach, and (c) low in the colon, liver, lung and heart) [35]. This variation may affect sesamol uptake and its clinical outcomes. Sesamol also has potential for being developed into skin whitening and cosmetic product [13,14]. Our study suggests that sesamol is most efficient for use in cells overexpressing LAT1 (e.g., as melanoma), and with less toxicity in cells expressing low LAT1 (e.g., non-cancerous epithelial cells).

In conclusion, we delineated the transport mechanism of sesamol. A greater mRNA expression of LAT1 over LAT2 was observed in SK-MEL-2 cells over Vero cells. Ours is the first study to report expression of LAT1 in the SK-MEL-2 cells. Sesamol can overcome limited passive diffusion across melanoma and non-cancerous epithelial membrane, using the specific LAT1 overexpressed in melanoma cells. The underlying intracellular transport pathway of sesamol indicates how sesamol engages its selective cytotoxicity in melanoma cells vs. normal cells with promising clinical benefit. This finding could be used to design and develop sesamol for skin treatment.

## 4. Materials and Methods

### 4.1. Materials

Sesamol (98% purity), 2-Amino-2-norbornanecarboxylic acid (BCH) (98% purity), L-dopa (98% purity), L-tyrosine (99% purity), and melphalan (95% purity) were purchased from Sigma-Aldrich (St. Louis, MO, USA). L-Leucine (99% purity) and L-alanine (99% purity) were purchased from TCI Chemicals (Tokyo, Japan). Methanol HPLC grade was purchased from RCI Labscan (Bangkok, Thailand). Dimethyl sulfoxide (DMSO) biological grade was purchased from PenReac AppliChem (Barcelona, Spain). The reagents and culture media including Dulbecco’s modified Eagle’s medium of high glucose (DMEM), Hank’s balanced salt solution (HBSS) without Ca^2^^+^ and Mg^2^^+^ supplemented, and penicillin and streptomycin were purchased from GIBCO^®^, Invitrogen (Grand Island, NY, USA). Fetal bovine serum (FBS) was purchased from GE Life Sciences (Parramatta, Australia). Sodium hydroxide (NaOH) and hydrochloric acid (HCl) were from RCI Labscan (Bangkok, Thailand).

### 4.2. Cell Lines and Cell Culture

Human melanoma (SK-MEL-2) cell line (CLS-Cell lines Service, Eppelheim, Germany) and African green monkey kidney epithelial (Vero) cell line (ATCC#CCL-81) were maintained in DMEM supplemented with 10% FBS, 100 units/mL of penicillin, and 100 µg/mL of streptomycin, at 37 °C in 5% CO_2_ atmosphere.

### 4.3. Cellular Uptake of Sesamol

SK-MEL-2 and Vero cells were seeded separately into 24-well plates at 2 × 10^5^ cells/well for 24 h. The media was removed, and the cells pre-incubated with HBSS pH 7.4 for 10 min. The cells were used for the following experiments.

#### 4.3.1. Time-Course Study

The cells were exposed to 1 mM (200 µL) sesamol and incubated at 37 °C at various times (0.5, 1, 3, 5, 10, and 15 min). The media was plated into 96-well plates prior to measuring the extracellular sesamol content by HPLC analysis. The cells were washed twice with ice-cold PBS, solubilized by methanol, and centrifuged at 2400× *g*, at 4 °C for 20 min. The precipitated protein was re-solubilized by 0.1 M NaOH and 25 µL was taken for protein determination using a BCA Protein Thermo Fisher Scientific Reagent Kit (Pierce Biotechnology, Rockford, IL, USA). The sesamol content in 20 µL of cell lysate was quantified using HPLC assay as described in the analytical procedure.

The cellular uptake of sesamol was determined based on the sesamol content in cells (nmol/mg protein) to medium (nmol/µL) (expressed as C/M ratio, µL/mg protein). The linear regression of the C/M ratio from 0.5 to 3 min was performed (*r^2^* > 0.90); to give the initial distribution volume (µL/mg protein) from the y-intercept, and the initial uptake clearance (µL/min/mg protein) from the slope.

#### 4.3.2. Uptake Kinetics

The cells were pre-incubated with HBSS pH 7.4 at 37 °C and at 4 °C both for 10 min. The cells were treated with sesamol (200 µL; range, 31.5 to 1,000 μM) and incubated under each condition for another 1 min, to exclude the influence of efflux or metabolism processes as per the previous report [36]. The uptake process at 37 °C represented the total uptake of sesamol in the cells, including both carrier-mediated transport and passive transport; whereas the uptake process at 4 °C represented mainly passive transport [37].

The uptake kinetics was fitted using Michaelis-Menten kinetics (non-linear least square kinetics model) (*r^2^* > 0.90) Equation (1) that allowed estimation of *V*_max_, *K*_m_, and *P*_d_ values.

*V* = (*V*_max_ × [*S*])/(*K*_m_ + [*S*]) + *P*_d_ × [*S*] (1)

*V* = uptake velocity at a certain amount of substrate (nmol/min/mg protein)

*V*_max_ = maximum uptake velocity (nmol/min/mg protein)

[*S*] = concentration of sesamol at extracellular compartment (µM)

*K*_m_ = concentration of sesamol at half of *V*_max_ (µM)

*P*_d_ = passive lipoidal diffusion uptake rate (µL/min/mg protein)

The rate to reach total uptake clearance of carrier-mediated transport was obtained from the ratio of *V*_max_ to *K*_m_ (µL/min/mg protein). The total uptake clearance of the process (µL/min/mg protein) was obtained from (*V*_max_ /*K*_m_) + *P*_d_.

#### 4.3.3. LAT1 Function

The respective competitive uptake between the LAT1 substrate (L-dopa) and the LAT1 inhibitor (BCH) or the other LAT1 substrates (i.e., L-tyrosine, L-leucine, L-alanine and melphalan) was performed. Sesamol was also investigated for its affinity to LAT1. The respective final concentration of L-dopa, BCH, L-tyrosine, L-leucine, L-alanine, melphalan, and sesamol was 0.1, 1, 1, 2, 2, 0.05 and 1 mM. Competitive uptake was performed for 10 min then the cells were washed twice with ice-cold PBS. The L-dopa was solubilized by 0.1 M HCl and the cell lysate (25 µL) was pipetted for protein determination. The remaining lysate was precipitated by centrifugation at 2400× *g* at 4 °C for 15 min. Intracellular and extracellular L-dopa was quantified by the HPLC assay. The L-dopa uptake was represented as the distribution volume of L-dopa in a cell to media ratio (C/M ratio) as calculated in Section 4.3.1.

#### 4.3.4. Competitive Uptake between Sesamol and the other LAT1 Substrates

Competitive inhibition of uptake of sesamol was done in both SK-MEL-2 and the Vero cells. Sesamol was co-incubated with the LAT1 inhibitor (BCH) or the LAT1 substrates (i.e., L-tyrosine, L-leucine, L-alanine, melphalan, or L-dopa) for 10 min in the SK-MEL-2 and Vero cells. The final concentration of sesamol, BCH, L-tyrosine, L-leucine, L-alanine, melphalan and L-dopa was 0.1, 1, 1, 2, 2, 0.05 and 1 mM, respectively. The cells were washed twice with ice-cold PBS, solubilized in methanol, and centrifuged at 2400× *g*, at 4 °C for 20 min. The precipitated protein was re-solubilized by 0.1 M NaOH prior to protein determination. The sesamol content was quantified using HPLC assay. The cellular uptake of sesamol was calculated as per the C/M ratio.

#### 4.3.5. Uptake Kinetics of Sesamol via LAT1-Mediated Transport

The cells were pre-incubated with the LAT1 substrates (either L-leucine or L-alanine,) or the LAT1 inhibitor (BCH) in HBSS pH 7.4 at 37 °C for 10 min. L-leucine and L-alanine were selected because of their respective high and low affinity for LAT1; compared to other L-type amino acids [38]. The various concentrations of sesamol were added to the cells for 1 min. The final concentration of sesamol ranged between 31.5 and 1000 μM, and for LAT1 substrates and inhibitor were 2 mM and 1 mM, respectively. The procedure followed the steps above. The uptake parameters—including *V*_max_ (nmol/min/mg protein), *K*_m_ (μM), and *P*_d_ (µL/min/mg protein)—were calculated using equation (1) and expressed as nmol/min/mg protein.

### 4.4. HPLC Analysis

Quantitative analysis of sesamol and L-dopa were performed using a Shimadzu HPLC (LC-2030C-3D) with a UV photodiode array detector (DAD) (Kyoto, Japan). The analysis was based on a BDS Hypersil C-18 column, (4.6 × 250 mm, 5 µm packing, Thermo Fisher Scientific, Waltham, MA, USA). The column temperature was set at 30 °C with a flow rate of 0.8 mL/min. The mobile phase for sesamol quantification was 70% methanol in water, vs. 20% methanol in water for L-dopa. The respective detection wavelength and elution time for sesamol and L-dopa was 297 nm and 280 nm and 4.2 and 4.5 min. The validated sesamol quantification conducted using sesamol spiked cell lysate with high selectivity and clear separation from the endogenous cell lysate as previously reported [39]. The % bias and % precision was determined within and between days, each was maintained below 6%.

### 4.5. Antiproliferative Effect

The SK-MEL-2 and Vero cells (at 5 × 10^4^ cells/well) were seeded separately into 96-well plates. After the cells were exposed to the tested compounds (200 µL) for 24 h, neutral red (50 µg/mL) (Sigma-Aldrich, St. Louis, MO, USA) was incorporated into the cells for 2 h. The cells were then washed with ice-cold PBS and lysed using 0.33% HCl in isopropanol. The absorbances of neutral red were measured at 540 nm with a reference wavelength of 660 nm using a UV-microplate reader (Perkin Elmer, Waltham, MA, USA). The % cell viability and 50% inhibitory concentration of cell viability (IC_50_) were calculated. The selective index was calculated from the ratio of the IC_50_ of Vero and SK-MEL-2 cells.

### 4.6. mRNA Expression of LAT1 and LAT2

Total RNA was extracted using the mRNA extraction kit (Vivantis Technologies, Selangor Darul Ehsan, Malaysia). The total RNA was reverse transcribed to cDNA using a Viva 2-step RT-PCR kit (Vivantis Technologies, Selangor Darul Ehsan, Malaysia). Beta-actin was used as the housekeeping gene. Quantitative PCR was performed using a sensiFAST^TM^ SYBR NO-ROX kit (Bioline USA Inc., Taunton, MA, USA) and determined by real-time PCR detection (Bio-rad, Hercules, CA, USA). The ΔΔCT method was used to compare mRNA expression between the SK-MEL-2 and Vero cells. The forward primer for LAT1 was 5′-ATC ATC CGG CCT TCA TCG CA-3′ and the reverse primer was 5′-CAC GCT GTA GCA GTT CAC GG-3′. The forward primer for LAT2 was 5′-CCA GGC ACC GAA ACA ACA CC-3′ and the reverse primer was 5′-AGC CGA TGA TGT TCC CTA CGA-3′. The forward primer for beta-actin was 5′-ACA GAG CCT CGC CTT TGCC-3′ and the reverse primer was 5′-GAT ATC ATC ATC CAT GGT GAG CTG G-3′.

### 4.7. Statistical Analysis

All experimental results were expressed as means with standard deviation. The difference between samples of independent observations were analyzed using a Kruskal Wallis nonparametric statistics. Statistical analyses were performed using SPSS 24.0 (SPSS Inc, IL, USA). *p* values below 0.05 were considered statistically significant. The non-linear least square, non-linear regression and sigmoidal Hill curve fit were performed using Datagraph version 4.3 (Visual data tools Inc, Chapel Hill, NC, USA).

## Figures and Tables

**Table 1:** Summary values of *V*_max_, *K*_m_, and *P*_d_ values of sesamol under each condition studied. Data are expressed as mean ± standard deviation of triplicates with two analytical runs. Different superscript letters in same column indicate significant differences between temperature samples (*p* < 0.05).

Conditions	Sesamol Uptake Kinetic Parameters					
	SK-MEL-2			Vero		
	*V*_max_ (nmol/min/mg)	*K*_m_ (µM)	*P*_d_ (µL/min/mg)	*V*_max_ (nmol/min/mg)	*K*_m_ (µM)	*P*_d_ (µL/min/mg)
37 °C	4.2 ± 0.1^a^	224.6 ± 19.3^a^	2.3 ± 0.2^b^	0.9 ± 0.0^a^	159.9 ± 18.4^a^	2.1 ± 0.1^a^
4 °C	0.3 ± 0.1^b^	26.8 ± 9.8^b^	2.7 ± 0.1^a^	0.4 ± 0.1^b^	66.1 ± 20.0^b^	2.4 ± 0.3^a^

**Table 2:** Summarized distribution volume and %inhibition of L-dopa and sesamol under each condition. Data are expressed as mean ± standard deviation of triplicates with two analytical runs. Different letters in the same column indicate significant differences between samples (*p* < 0.05).

**Compounds**	**Percent Inhibition of L**-**Dopa Distribution Volume** (**L**-**Dopa Distribution Volume; µL**/**mg Protein**)	
	**SK**-**MEL**-**2**	**Vero Cells**
Control	0% (370.6 ± 13.0)^a^	0% (155.6 ± 17.8)^a^
1 mM BCH	82% (67.3 ± 14.0)^c^	69% (47.9 ± 3.7)^b,c,d^
1 mM L-tyrosine	72% (105.0 ± 5.2)^c^	69% (48.7 ± 11.0)^c,d^
2 mM L-leucine	75% (94.1 ± 13.5)^c^	75% (39.1 ± 2.3)^d^
2 mM L-Alanine	67% (121.2 ± 3.6)^b^	35% (101.3 ± 4.0)^a,b^
0.05 mM Melphalan	47% (196.6 ± 20.3)^a^	39% (95.5 ± 11.2)^a,b,c^
1 mM Sesamol	44% (207.6 ± 31.5)^a^	33% (103.9 ± 9.2)^a,b^
**Compounds**	**Percent Inhibition of Sesamol Distribution Volume** (**Sesamol Distribution Volume; µL**/**mg Protein**)	
	**SK**-**MEL**-**2**	**Vero Cells**
Control	0% (23.2 ± 2.1)^a^	0% (17.9 ± 1.7)^a^
1 mM BCH	55% (10.3 ± 0.9)^c,d^	42% (10.4 ± 1.9)^c^
1 mM L-tyrosine	75% (5.8 ± 0.2)^e^	41% (10.5 ± 0.7) ^c^
2 mM L-leucine	50% (11.7 ± 0.7)^c^	23% (13.8 ± 1.4)^b^
2 mM L-Alanine	44% (13.1 ± 0.3)^b^	25% (13.3 ± 2.0)^b,c^
0.05 mM Melphalan	60% (9.4 ± 0.7)^c,d^	35% (11.7 ± 0.7)^b,c^
1 mM L-Dopa	68% (7.4 ± 0.8)^d^	40% (10.8 ± 2.0)^b,c^

**Table 3:** Summarized values of *V*_max_, *K*_m_, and *P*_d_ values of sesamol under each condition studied. Data are expressed as mean ± standard deviation of triplicates with two analytical runs. Different letters in the same column indicate significant differences between samples (*p* < 0.05).

Compounds	Sesamol Uptake Kinetic Parameters					
	SK-MEL-2			Vero		
	*V*_max_ (nmol/min/mg)	*K*_m_ (µM)	*P*_d_ (µL/min/mg)	*V*_max_ (nmol/min/mg)	*K*_m_ (µM)	*P*_d_ (µL/min/mg)
Sesamol	4.2 ± 0.1^a^	224.6 ± 19.3^a^	2.3 ± 0.2^b^	0.9 ± 0.0^a^	159.9 ± 18.4^a^	2.1 ± 0.1^a^
Sesamol + BCH	0.2 ± 0.0^d^	6.0 ± 1.1^d^	0.5 ± 0.1^d^	0.3 ± 0.1^b^	38.2 ± 10.2^b^	0.4 ± 0.2^b^
Sesamol + L-Leucine	0.6 ± 0.2^c^	62.1 ± 20.8^b^	1.7 ± 0.2^c^	0.4 ± 0.1^b^	8.6 ± 2.4^c^	2.5 ± 0.4^a^
Sesamol + L-Alanine	1.4 ± 0.2^b^	34.9 ± 3.5^c^	2.9 ± 0.0^a^	0.7 ± 0.0^b^	5.6 ± 1.7^c^	2.4 ± 0.0^a^

**Table 4:** Summarized values of IC_50_ of sesamol and melphalan under each condition studied. Data are expressed as mean ± standard deviation of three replicates. Different letters indicate significant differences between samples (*p* < 0.05).

Compounds	IC_50_ (mM)		Selective Index
	SK-MEL-2	Vero Cells	
Sesamol	2.0 ± 0.1^b^	6.7 ± 0.2^a^	3.4
Sesamol + 1 mM BCH	3.0 ± 0.0^a^	4.8 ± 0.1^b^	1.6
Melphalan	0.1 ± 0.0^d^	0.6 ± 0.3^b^	6.0
Melphalan + 1 mM BCH	0.2 ± 0.0^c^	0.5 ± 0.1^b^	2.5

## Supplementary Materials

The following are available online at https://www.mdpi.com/1420-3049/24/21/3869/s1, Figure S1: Interaction of sesamol with human LAT1-4F2hc chain.
