# Supplementary material for: Role of L-Type Amino Acid Transporter 1 (LAT1) for the Selective Cytotoxicity of Sesamol in Human Melanoma Cells
Source: Molecules. 2019 Oct 27;24(21):3869. doi: 10.3390/molecules24213869 (PMC6865181; doi:10.3390/molecules24213869)
Supplement: Supplementary file 1 [file molecules-24-03869-s001.pdf]

Supplementary Materials

Article

Role of L-Type Amino Acid Transporter 1 (LAT1) for the Selective Cytotoxicity of Sesamol in Human Melanoma Cells

Tarapong Srisongkram [1], Natthida Weerapreeyakul [2,3,*], Jussi Kärkkäinen [4] and Jarkko Rautio [4]

[1] Graduate School (in the program of Research and Development in Pharmaceuticals), Faculty of Pharmaceutical Sciences, Khon Kaen University, Khon Kaen 40002, Thailand; tarapong.sri@gmail.com

[2] Division of Pharmaceutical Chemistry, Faculty of Pharmaceutical Sciences, Khon Kaen University, Khon Kaen 40002, Thailand; natthida@kku.ac.th

[3] Human High Performance and Health Promotion (HHP&HP) Research Institute, Khon Kaen University, Khon Kaen 40002, Thailand; natthida@kku.ac.th

[4] School of Pharmacy, University of Eastern Finland, Kuopio 70211, Finland; jussi.karkkainen@uef.fi (J.K.); jarkko.rautio@uef.fi (J.R.)

* Correspondence: natthida@kku.ac.th; Tel.: +66-432-023-78

Supplementary Figure S1

(A) (B)

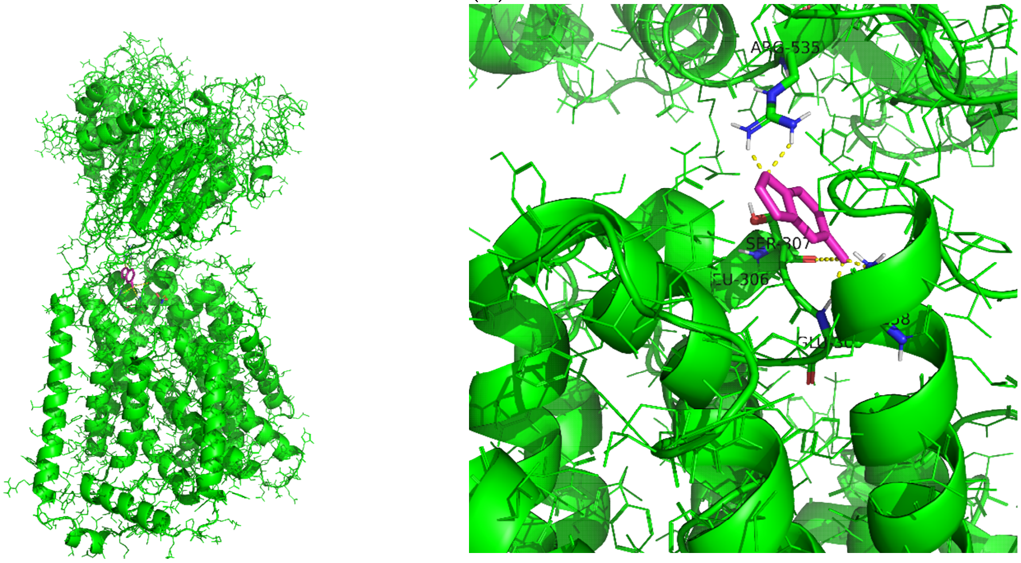

Figure S1. Interaction of sesamol with human LAT1-4F2hc chain. (**A**) Predicted interaction site between sesamol and LAT1-4F2hc protein. (**B**) Hydrogen bonds of hydroxyl group of sesamol with serine 307, glutamic acid 309, and lysine 158 of LAT1 transmembrane and the hydrogen bonds of oxygen in the benzodioxol ring of sesamol with arginine 535 of 4F2hc domain. Blue and red atom color indicates a nitrogen and oxygen atom, respectively.
